# Community-based health care is an essential component of a resilient health system: evidence from Ebola outbreak in Liberia

**DOI:** 10.1186/s12889-016-4012-y

**Published:** 2017-01-17

**Authors:** Kendra Siekmans, Salim Sohani, Tamba Boima, Florence Koffa, Luay Basil, Saïd Laaziz

**Affiliations:** 1 HealthBridge, 1 Nicholas Street, Suite 1004, Ottawa, ON K1N 7B7 Canada; 2 Canadian Red Cross Society, 170 Metcalfe Street, Ottawa, ON K2P 2P2 Canada; 3 Ministry of Health and Social Welfare, P. O. Box 10–9009, 1000, Monrovia, 10 Liberia; 4 Liberia Red Cross Society, 107 Lynch Street, 1000, Monrovia, 20 Liberia

**Keywords:** Liberia, Integrated community case management, Diarrhea, Pneumonia, Ebola, Health system, Community health worker

## Abstract

**Background:**

Trained community health workers (CHW) enhance access to essential primary health care services in contexts where the health system lacks capacity to adequately deliver them. In Liberia, the Ebola outbreak further disrupted health system function. The objective of this study is to examine the value of a community-based health system in ensuring continued treatment of child illnesses during the outbreak and the role that CHWs had in Ebola prevention activities.

**Methods:**

A descriptive observational study design used mixed methods to collect data from CHWs (structured survey, *n* = 60; focus group discussions, *n* = 16), government health facility workers and project staff. Monthly data on child diarrhea and pneumonia treatment were gathered from CHW case registers and local health facility records.

**Results:**

Coverage for community-based treatment of child diarrhea and pneumonia continued throughout the outbreak in project areas. A slight decrease in cases treated during the height of the outbreak, from 50 to 28% of registers with at least one treatment per month, was attributed to directives not to touch others, lack of essential medicines and fear of contracting Ebola. In a climate of distrust, where health workers were reluctant to treat patients, sick people were afraid to self-identify and caregivers were afraid to take children to the clinic, CHWs were a trusted source of advice and Ebola prevention education. These findings reaffirm the value of recruiting and training local workers who are trusted by the community and understand the social and cultural complexities of this relationship. “No touch” integrated community case management (iCCM) guidelines distributed at the height of the outbreak gave CHWs renewed confidence in assessing and treating sick children.

**Conclusions:**

Investments in community-based health service delivery contributed to continued access to lifesaving treatment for child pneumonia and diarrhea during the Ebola outbreak, making communities more resilient when facility-based health services were impacted by the crisis. To maximize the effectiveness of these interventions during a crisis, proactive training of CHWs in infection prevention and “no touch” iCCM guidelines, strengthening drug supply chain management and finding alternative ways to provide supportive supervision when movements are restricted are recommended.

**Electronic supplementary material:**

The online version of this article (doi:10.1186/s12889-016-4012-y) contains supplementary material, which is available to authorized users.

## Background

Trained community health workers (CHW) help ensure equitable access to essential primary health care services in contexts where the health system lacks the capacity to adequately deliver these services [1–4]. While demonstrated in stable settings, the role of this cadre of health workers is not well documented in contexts where short- or long-term crises have undermined health system function.

Liberia’s first two cases of Ebola Virus Disease were confirmed on March 30, 2014 and the disease rapidly spread through several counties in the country. By May 9, 2015, there were a total of 10,666 cases (3151 confirmed, 1879 probable and 5636 suspected cases) reported in Liberia, including 4806 deaths [5]. The national health system in Liberia, characterized by a lack of adequately qualified health staff and medical supplies before the outbreak [6], was further weakened by the strain of the Ebola crisis, resulting in a major disruption in the delivery of maternal and child health services [7–9]. Although the public health impact of the Ebola outbreak is difficult to quantify, primary health care provision was nearly non-existent for a period of time and many children did not receive treatment for malaria, pneumonia and diarrhea [10].

Prior to the Ebola outbreak, the Liberia Ministry of Health and Social Welfare (MoHSW) developed the National Strategy and Policy for Community Health Services in an effort to increase access to maternal and child health services through health promotion and case management [11]. Integrated community case management (iCCM) of child illnesses was introduced in 2010, with County Health Teams responsible for supervising the general community health volunteers (Liberian term used for CHW; hereafter referred to as CHW) working at the community level in each health facility’s catchment area. However, iCCM implementation in Liberia has faced various challenges, including problems in drug supply, supervision, and lack of volunteer incentives. Implementation continues to be dependent on availability of development partner funding.

In May 2012, Liberian National Red Cross Society (LNRCS) initiated a maternal, newborn and child health (MNCH) project with the aim of improved access to primary health care services for 40,900 people in 43 hard-to-reach communities (>5 km from nearest health facility) in Gbarpolu (pop 83,758), Bomi (pop 82,036) and Grand Gedeh (pop 126,146) counties, covering 14%, 21% and 9% of each county’s population respectively [12]. These three counties were chosen based on their low coverage for maternal and child health interventions, requests from the MoHSW, remote locations and limited presence of other NGOs. To implement the Community Health Services Strategy, the project worked in coordination with the respective County Health Teams to train, equip and supervise CHWs to deliver iCCM. As recommended by the national protocol, CHWs were given approximately 50 USD quarterly in non-monetary incentives (rice, oil, bouillon cubes).

Despite serious disruptions in health system function during the Ebola outbreak, the project’s community-based work by CHWs continued, generating interest in the role that CHWs played in mitigating the negative impact of the Ebola crisis on child health. This study assesses the value of a community-based health system in ensuring continued delivery of essential health services in the context of a national crisis (Ebola epidemic) in three Liberian counties. A secondary objective is to document the role that CHWs had in relation to Ebola prevention activities.

## Methods

The study gathered and analyzed new and existing data from project communities across three counties that differed widely in their experience of the outbreak. Bomi County, with a high population density (109 persons per square mile, PPSM [12]) and bordering Montsserado County, one of the epicenters of the outbreak, had 139 confirmed cases; the more rural Gbarpolu County (22 PPSM) and Grand Gedeh County (31 PPSM) had only 16 and 3 confirmed cases, respectively [5]. A descriptive observational design was used, with a mixed methods approach to data collection using five sources. Triangulation of results was used to establish the adequacy of the evidence provided and the extent to which the results could meet the study’s objectives.

Routine monitoring data from individual CHW registers (*N* = 92; see Additional file 1) recording the number of children treated and referred for diarrhea, pneumonia and malaria by month as well as information on medicine stocks were gathered by project staff for the period of January 2014 to March 2015. These data were used to assess CHW delivery of iCCM interventions before, during and after the Ebola crisis. Treatment for malaria cases was excluded from the analysis because this did not start until late December 2014.

Immunization coverage data from expanded program of immunization (EPI) registers and number of children under 5 years treated for diarrhea, pneumonia and malaria from Outpatient Department or Child Health Registers were obtained from government health facilities in the project catchment areas (six clinics and one government hospital in Bomi County, two clinics in Gbarpolu County, and three clinics and one health center in Grand Gedeh County) to assess service delivery for the period of January 2013 to February 2015.

Focus group discussions were held with two groups of CHWs purposively selected from project communities in all three counties (Bomi *n* = 8, Gbarpolu *n* = 4, Grand Gedeh *n* = 4). The selection criteria used were: at least two female CHWs per group (65% of the project CHWs are male) and diverse representation based on community remoteness and Ebola outbreak intensity. Participants gathered in a meeting room for the discussion led by the first author via Skype audio, with local support from a project staff member. The discussions were facilitated in English and recorded for transcription purposes.

A structured survey was administered to a random sample of 60 CHWs (out of 100 CHWs involved in the project) to assess their perceptions of the impact that the Ebola crisis had on access to essential health services in their communities and their role during the crisis. [See Additional file 2 for a copy of the survey questionnaire and Additional file 3 for a copy of the data.] Selected CHWs were interviewed in-person (Bomi and Gbarpolu counties) or by telephone (Grand Gedeh County).

Key informant interviews were conducted in person and by telephone using a standard questionnaire with open-ended questions with the county health officers and health facility staff responsible for supervising CHWs, as well as with project staff, to document their perceptions of the role that community-based health service delivery had on access to essential health services during the Ebola crisis.

### Data management and statistical analysis

Project monitoring data and health facility coverage data were entered in Microsoft Excel and analyzed for service delivery time trends. The CHW survey data were entered and analyzed in Epi Info 7 (CDC, Atlanta, GA). Differences across county were tested using Fisher’s exact test for proportions and one-way analysis of variance for means. A reflexive, iterative process of data management was used to analyse the content of the interviews and the focus group discussions [13]. Following a review of written interview notes and confirming their accuracy with the audio recording, content analysis was used to elicit common themes. Analysis documented trends over time in service delivery and sought to understand the reasons for the continuation and/or discontinuation of iCCM, using pre-specified categories of analysis and associated questions (see Additional File 4).

## Results

**Fig. 1:**
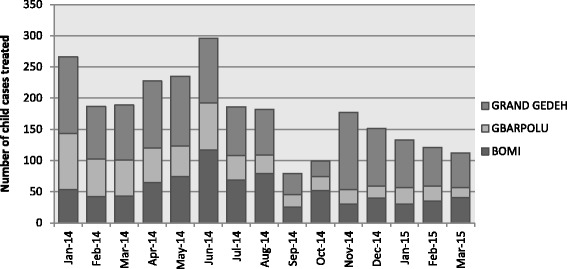
Number of child diarrhea and ARI cases treated by CHWs in project communities between January 2014 and March 2015, by county (Source: Community CHW Registers)

Coverage for community-based treatment of child diarrhea and pneumonia in project communities is shown in Fig. 1. Results show continuous treatment of children in project areas by CHWs before, during and after the Ebola outbreak, with a decrease in the total number of cases treated during the height of the outbreak (September and October 2014). Prior to the outbreak and until July 2014, over 50% of CHW registers recorded at least one treatment per month for diarrhea or ARI. This decreased to 42, 28 and 34% in August, September and October, respectively, and then increased to earlier levels by November.

**Fig. 2:**
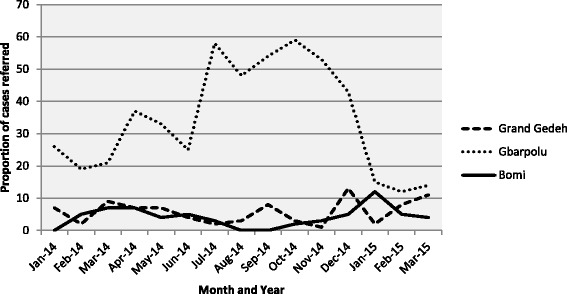
Proportion of child diarrhea and ARI cases referred by CHWs in project communities between January 2014 and March 2015, by county (Source: Community CHW Registers)

Referral rates by CHWs in Gbarpolu County were higher than the other two counties and increased dramatically between July and December 2014 (see Fig. 2). However, in Grand Gedeh and Bomi Counties, referrals were <10% for most of the period under review, with zero cases referred in August and September 2014 in Bomi County.

When asked in the survey, most CHWs (73%) reported a decrease in the number of cases they consulted during the outbreak (Table 1). Approximately two thirds said the reason was fear of contracting Ebola while seeking treatment for their children’s diarrhea or pneumonia. In contrast, 15% of CHWs reported an *increase* in number of consultations due to the family’s fear of seeking care from a health facility due to risk of Ebola infection and the lack of staff or medicines at the nearest health facility.

**Table 1:** CHW characteristics and activities

Indicator	Bomi	Gbarpolu	Grand Gedeh	Overall
N	17	18	25	60
Male, n (%)	14 (82)	16 (89)	18 (72)	48 (80)
Mean age, years	37	36	33	35
Any suspected or confirmed cases of Ebola infection in their community, n (%)	2 (12)	5 (28)	-	7 (12)*
Anyone died from Ebola in their community, n (%)	2 (12)	-	-	2 (3)
Observed trend in CHW services during Ebola outbreak compared to before the outbreak, n (%)				
Stayed the same	3 (18)	2 (11)	2 (8)	7 (12)
Increased	3 (18)	2 (11)	4 (16)	9 (15)
Decreased	11 (65)	14 (78)	19 (76)	44 (73)
Drugs available during the Ebola crisis, n (%)^a^				
Diarrhea	11 (65)	8 (44)	19 (76)	38 (63)
Pneumonia	7 (41)	8 (44)	16 (64)	31 (52)
Drugs available currently, n (%)				
Diarrhea	10 (59)	6 (33)	23 (92)	39 (65)*
Pneumonia	4 (24)	1 (6)	17 (68)	22 (37)*
Malaria	7 (41)	17 (94)	23 (92)	47 (78)*
Level of HF functioning during outbreak, mean (median) (1 = non-functional; 5 = fully functional)	1.6 (1)	3.0 (4)	3.1 (2)	2.7 (2)*
Supervision received during Ebola crisis, n (%)	15 (88)	18 (100)	24 (96)	57 (95)
Red Cross project staff	12 (71)	17 (94)	21 (84)	50 (83)
MOH	6 (35)	6 (33)	12 (48)	24 (40)
Other NGO	-	-	7 (28)	7 (12)
Received training on No-Touch Policy, n (%)	16 (100)	18 (100)	24 (96)	58 (98)
Who provided training on No-Touch Policy, n (%)				
Red Cross project	11 (69)	18 (100)	23 (92)	52 (88)*
MOH Staff	6 (38)	7 (39)	7 (28)	20 (34)
Other NGO	2 (13)	1 (6)	17 (68)	20 (34)*
Used No-Touch guidelines when assessing sick children, n (%)	13 (76)	18 (100)	24 (96)	55 (92)
Contact from MOH/local health facility during the Ebola response^b^, n (%)	11 (65)	12 (67)	22 (88)	45 (75)
Engaged in Ebola prevention education to community, n (%)	17 (100)	18 (100)	25 (100)	60 (100)
Support received to provide Ebola prevention education activities, n (%)				
Red Cross project staff	12 (71)	13 (72)	21 (84)	46 (77)
MOH Health Staff	3 (18)	4 (22)	5 (20)	12 (20)
Community Health Committee	6 (35)	4 (22)	-	10 (17)*
Other NGO	4 (24)	7 (39)	16 (64)	27 (45)*

* *p* < 0.05 for Fisher’s exact test of differences in proportions across County or Anova f-test of differences in means across County

^a^Malaria drugs had not yet been distributed to CHWs at the time of the Ebola outbreak

^b^80% reported the purpose of the visit was to ask the CHW to create awareness and provide Ebola prevention education

In focus group discussions, some CHWs reported stopping treatment of sick children during the Ebola outbreak due to the government’s directives for people to not touch others, especially sick people. One county health team member stated that CHWs were instructed to refer all cases so that the health facility could pick up cases of Ebola. Given the difficulty in assessing a sick child without touching them and uncertainty regarding the cause of fever and diarrhea, the majority of CHWs reported direct referral of all sick children to the nearest health facility.

“*We were told not to touch, so during Ebola, I did not treat. When someone brought a child, I did not know if they had Ebola or not, so I referred.*” (CHW, Bomi County)

However, CHWs reported having to advocate strongly for caregivers to take children to the health facility due to widespread fear of Ebola infection. Furthermore, patients were often not treated there but referred to the county hospital.

*“…some would have reason, they would go. Some would just stay home. They would say ‘if the child die, let the child die, but we will not go to the hospital.’” (CHW, Grand Gedeh County)*

Another reported barrier to community-based treatment was a disruption in drug supply. Although individual CHW drug records and survey results showed that almost two thirds of CHWs had diarrhea drugs and over half had pneumonia drugs during the outbreak, many CHWs in the focus group discussions indicated that their drug supply ran out. In Grand Gedeh County, where CHWs obtained medicines directly from the nearest health facility, medicine stocks were available during the outbreak but CHWs reported inability to access them due to travel restrictions and facility closures. In the other counties, stock outs at the Red Cross level affected access.

The rating by CHWs of health facility function during the Ebola outbreak varied significantly by county. In Bomi County, 12 of 17 CHWs (71%) rated their health facility as non-functional. However, in Gbarpolu County, one third of CHWs rated their nearest facility as non-functional during the outbreak and 50% as moderately functional. In Grand Gedeh, 13 of the 25 CHWs rated the health facility as low functioning and one-third rated it as fully functional.

**Fig. 3:**
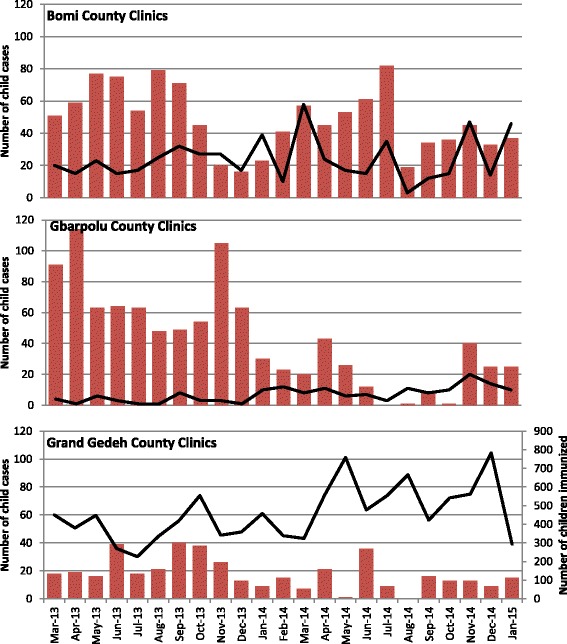
Government health facility monthly monitoring data for immunizations (black line and right vertical axis, source: EPI Register) and treatment of diarrhea and pneumonia in children under 5 years of age (red columns, left vertical axis, source: OPD/Child Health Register). Data available from 4 clinics in Bomi County; 2 clinics in Gbarpolu County; 4 clinics in Grand Gedeh

Based on routine monitoring data collected from the health facilities themselves, there is some evidence of a decrease in service provision during the peak of the crisis (see Fig. 3), although not to the scale that was anecdotally reported. Government health facility staff explained that a lack of personal protective equipment and fear of Ebola infection resulted in health facility workers leaving their posts.

**Fig. 4:**
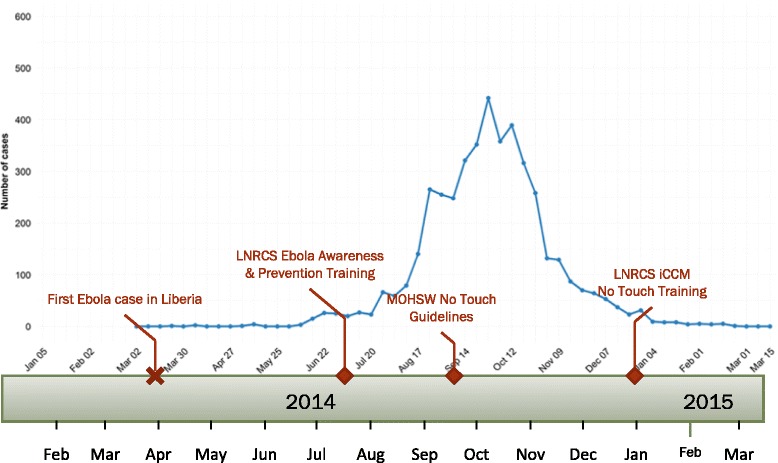
Timeline showing peak in weekly Ebola virus disease cases reported nationally from Liberia and important project events (note: data are laboratory confirmed cases reported by the Liberian Ministry of Health; adapted from [27])

Interviews with CHWs and project staff revealed that the government circulated “No Touch iCCM” guidance at the height of the outbreak (September 2014, see Fig. 4), based on WHO/UNICEF guidelines for a revised implementation of iCCM in the context of Ebola [14]. Although project staff promptly circulated the information to CHWs, formal training of CHWs on “No Touch iCCM” took place in December and January, as part of training on treatment of child malaria. In focus group discussions, several CHWs emphasized that this training gave practical guidance and renewed confidence in assessing and treating sick children. All but two of the CHWs surveyed had been trained and 55 of 60 (92%) reported using these guidelines when treating sick children.

All sources confirmed that CHWs were asked by the MOHSW, LNRCS and other NGOs to communicate Ebola awareness and prevention messages. This became their primary activity during the outbreak; 78% of CHWs surveyed conducted house-to-house visits and 50% used community meetings to disseminate messages. In Bomi and Gbarpolu counties, two CHWs were asked to do active case finding or contact tracing and two were asked to help set up a community Ebola task force. The role of the Community Health Committee, as reported by CHWs, was similar, except for four communities where the Committee was reported to have done nothing.

The presence of an ongoing project with staff and funding to support community-based health service delivery was considered to have mitigated the negative impact of the Ebola outbreak in project communities. The LNRCS was credited by 90% of surveyed CHWs with providing Ebola awareness and prevention training and support, as well as dissemination of the “No Touch” guidelines and in-depth training on their use.

## Discussion

The global strategy for Women’s, Children’s and Adolescents’ Health and the Sustainable Development Goals are prompting a shift toward strengthening community health platforms to deliver a comprehensive package of services, including health promotion and prevention as well as curative services, with emphasis on linking these community services to first level facilities to support the continuum of care (D. Kasungami, personal communication). The results of our assessment of community-based health service delivery during the Ebola outbreak in Liberia demonstrate its strengths and weaknesses. One strength is the presence of community actors who are already engaged in health promotion and basic treatment services. Investments in community level health systems help to build a community’s resilience by providing training, equipment and essential medicines to community-based providers for basic health services [15]. In times of crisis, community health workers can quickly disseminate disease prevention messages [16], identify cases for referral and provide follow-up care. The presence of CHWs trained in iCCM and medicine supplies in project communities helped to ensure continued access to lifesaving treatment for child pneumonia and diarrhea. Being located in the community was particularly important during the Ebola outbreak due to imposed limits on population movement. These personnel can also play an essential role now in the recovery process, promoting vaccination, encouraging attendance at health care facilities, and distributing medication [10]. A high level of community engagement with the public health system helped buffer the negative impact of a crisis. Consultation between health facility personnel and CHWs before the outbreak resulted in more effective risk communication and community action to reduce risks during the outbreak [17]. In a climate of distrust, where health facility workers were reluctant to treat patients, sick people were afraid to self-identify, caregivers were afraid to take children to the clinic, and pregnant women were often turned away from health facilities due to the high risk that they posed [18, 19], CHWs were a trusted source of advice, treatment for child illnesses and Ebola prevention education. These findings reaffirm the value of recruiting and training local workers who are trusted by the community and understand the social and cultural complexities of this relationship [15, 20, 21].

However, the role of CHWs also placed them at increased risk of becoming infected with the Ebola virus [9]. Although “no touch” iCCM guidelines sought to maintain continuity of services at the community level in a safe manner, the timing of this guidance and its associated training were very late. More proactive action is recommended to train all CHWs in basic infection control measures and provide them with the necessary protective equipment [8, 14, 20].

The breakdown in the referral system highlights the difficulty of implementing iCCM in the context of a major crisis. Although the “no touch” iCCM guidelines recommended continued referrals, this became increasingly difficult to implement as local clinics closed and families refused to comply. Inconsistencies observed between CHW self-reported referral practices and the data recorded in their registers are likely due to variation over time in referrals that corresponded with level of function of the nearest health facility as well as medicine stocks. In Bomi County, where CHWs reported low levels of health facility function during the outbreak, there were no referrals recorded in CHW registers for August and September. In Gbarpolu County, where health facility function remained moderate, referrals increased during this period. These results demonstrate that CHWs can play an important role in encouraging health care-seeking behaviours, even in the context of a major national crisis, but also that iCCM programs must consider the value and utility of referrals in contexts where health facilities are low- or non-functioning.

Supply chain management is another critical component for successful iCCM implementation; drug stock outs reduce program effectiveness and acceptability [22, 23]. The interruption in supply of medicines was identified by CHWs as one contributor to the decrease in diarrhea and pneumonia cases treated, despite project efforts before the outbreak to establish accessible supplies. During the outbreak, monitoring of drug stocks was done on an ad hoc basis by project staff. Without close supervision it is not known to what extent medicines were used for other purposes (e.g., treatment of adults). Improving product availability at resupply points as well as the supply chain knowledge and capacity among CHWs and their supervisors is recommended [22].

Routine supervision of CHWs by County Health Teams also declined during the Ebola crisis due to closure of health facilities, restrictions on movement and Ebola-related activities increasing in priority. Regular supervision ensures the quality of iCCM interventions [24–26] and its absence was evident in several respects during the crisis, including observed inconsistencies in treatments and referrals recorded in CHW registers and difficulties reported by CHWs in diagnosing patients without touching them. Alternative means of providing supportive supervision from a distance, such as through cell phone calls, are recommended to overcome barriers to in-person follow-up.

Our assessment demonstrates that the MNCH project’s human and financial resources were helpful in providing Ebola awareness and prevention training and support to CHWs, but they could not offset the full impact of the crisis. In particular, the closure and lower level of function of primary health care facilities during the outbreak still had a negative impact on access to health services. This is consistent with learning shared by the global iCCM Task Force that iCCM implementation is stronger when aligned to a functioning health system to ensure supportive supervision, drug supplies, and a functioning referral system between communities and facilities (D. Kasungami, personal communication).

### Limitations

Several limitations in our study may have influenced the results, including the involvement of project staff in data collection and interviews that may have biased responses. Efforts were made to minimize this bias by the careful explanation of the study purpose and assuring respondents that their input was not going to be used in any way to evaluate their performance. Having an external evaluator on the assessment team, collecting both subjective and objective data, and triangulating results were expected to help reduce the bias at the analytical and reporting levels. The accuracy of data from CHW registers was impossible to assess due to apparent underreporting by CHWs of treatments given during the outbreak. However, any additional treatments given would only strengthen the conclusions. Due to travel restrictions, the facilitation of focus group discussions was conducted using audio methods on less-than-optimal internet and cell phone connections. While this limited the facilitator’s ability to observe non-verbal communication among participants and gauge the level of agreement with statements made, the in-country presence of two project staff helped with participation and clarification of questions/responses.

The study was conducted in predominantly rural areas that were not as hard hit by the Ebola outbreak as some urban and peri-urban areas in Liberia. Furthermore, the communities were directly benefiting from an MNCH project and therefore had access to additional resources such as information on the outbreak, inputs to assist with Ebola education and prevention efforts, and periodic contact with project staff. This may limit the external validity of our results to other similar contexts in Liberia and other LMIC.

## Conclusion

Although the public health impact of the Ebola outbreak is yet to be fully appreciated, there is consensus that a major disruption in health facility service delivery in Liberia put the lives of many children at risk, not due to Ebola infection but rather lack of treatment for common childhood illnesses such as malaria, pneumonia and diarrhea. The results of this study demonstrate that continued investment in the Community Health Strategy in Liberia is warranted in terms of its contribution to building the resilience of communities during a crisis and ensuring prompt access to life-saving treatment for children at the community level. Despite many constraints and considerable uncertainty regarding their personal safety, CHWs were instrumental in providing both curative and preventive actions during the Ebola outbreak. Further strengthening of training in infection prevention and “no touch” iCCM guidelines, drug supply chain management, and alternative methods for supportive supervision during a crisis are recommended in this post-Ebola context.

## Additional files

Additional file 1: gCHV Community Register Data. This file provides the raw data and summary data of CHW community registers in the LNRCS MNCH project communities. (XLS 299 kb)
Additional file 2: Questionnaire for gCHVs working in iCCM during the Ebola crisis. This is a copy of the survey questionnaire administered to CHWs. (DOCX 25 kb)
Additional file 3: gCHV Survey Data. This is the raw data collected during the CHW survey. (XLSX 32 kb)
Additional file 4: Categories and associated questions guiding the data analysis process. This is a summary of the broad categories and their associated questions used to analyze the data. (DOCX 16 kb)

## Abbreviations

CHW: Community health worker
EPI: Expanded program of immunization
iCCM: Integrated community case
LNRCS: Liberian National Red Cross Society
MNCH: Maternal, newborn and child health
MoHSW: Ministry of Health and Social
NGO: Non-government organization
