# Supplementary material for: Community-based health care is an essential component of a resilient health system: evidence from Ebola outbreak in Liberia
Source: BMC Public Health. 2017 Jan 17;17:84. doi: 10.1186/s12889-016-4012-y (PMC5240441; doi:10.1186/s12889-016-4012-y)
Supplement: Additional file 2: — Questionnaire for gCHVs working in iCCM during the Ebola crisis. This is a copy of the survey questionnaire administered to CHWs. (DOCX 25 kb) [file 12889_2016_4012_MOESM2_ESM.docx]

Questionnaire for gCHVs Working in iCCM During the Ebola Crisis

| 1 | For how long have you been a government Community Health Volunteer? | Number of years _____ months ____ |
| --- | --- | --- |
| 2 | What type of training have you completed? | - iCCM - Red Cross Community-based Health and First Aid (CBHFA) |
| 3 | For which diseases have you been trained on for treatment of children under 5 years of age? | - Diarrhea - Pneumonia - Malaria - Malnutrition - None 🡺 Q#11 |
| 4 | If you compare the number of cases of all types that you provided services for before the Ebola outbreak and during the epidemic, would you say it remained the same, increased or decreased? | - Same - Increased 🡺 Q#5 - Decreased 🡺 Q#6 |
| 5 | *(If increased)* Why was there an increase in the number of cases you saw?  *Do not read the answers; check all applicable answers* | - Fear of going to health facilities due to Ebola infection - No staff at Health facilities - No medicine at Health facilities - Health facilities were closed - Health facility is too far - No transport or money to pay for transport to go to health facility - Community values gCHV services - Other (please state): ________________________________________________________ |
| 6 | *(If decreased)* Why do you think there was a decrease in the number of cases you saw?  *Do not read the answers; check all applicable answers* | - People afraid to seek treatment due to fear of Ebola infection - People choosing to consult traditional healers - People choosing to treat at home - Community did not trust CHV - Other (please state): ___________________________________________________ |
| 7 | During the Ebola crisis, did you have enough drugs for treating children with... | Diarrhea ❑ Yes ❑ No  Pneumonia ❑ Yes ❑ No  Malaria ❑ Yes ❑ No |
| 8 | Do you currently have drugs for treating children with... | Diarrhea ❑ Yes ❑ No  Pneumonia ❑ Yes ❑ No  Malaria ❑ Yes ❑ No |
| 9 | Who provides you with the drugs?  *Do not read the answers* | - Red Cross project - Health facility; MOH - Other NGO - Other - Don’t know |
| 10 | During the Ebola crisis, when you identified a child whose illness required the care from a trained health care provider (e.g. nurse or doctor), what did you advise the family to do? | - Nothing - Advised them verbally to go to the hospital or clinic - Referred them to the hospital or clinic (with a written paper) - Informed Community Health Committee - Informed my supervisor or the Red Cross project staff - Other |
| 11 | During the Ebola crisis, on a scale of 1 to 5, what was the level of functioning of the health facility nearest to your community?  *Functional* = trained staff present, drugs available, basic services being provided  *Non-functional* = no trained staff, no drugs, no services | **1 2 3 4 5**  Non- Fully  functional functional |
| 12 | During the Ebola crisis, did you receive any supervision visits? | - Yes ❑ No |
| 13 | If yes, who conducted the supervision visit? | - Health worker (MOH) - Red Cross Project staff - Other NGO - Other |
| 14 | Are you aware of the No-touch Policy issued by the Ministry of Health when diagnosing children during the Ebola epidemic? | - Yes ❑ No |
| 15 | When did you first hear about the No-touch Policy? | (Month)_______________, 2014 |
| 16 | How did you find out about the No-touch Policy?  *Do not read the answers; check all applicable answers* | - MOH staff - Red Cross project - Community Health Committee - Other NGO - Media (radio, TV, posters) - Other _________________ |
| 17 | Did you receive training on the No-touch Policy? | - Yes ❑ No |
| 18 | If yes, who trained you on the No-touch Policy?  *Do not read the answers; check all applicable answers* | - MOH staff - Red Cross project - Other NGO - Other _________________ |
| 19 | Did you use the No-touch guidelines when assessing and diagnosing sick children? | - Yes ❑ No |

| 20 | If no, why did you not use the No-touch guidelines? | - Not convenient to use - Fear of making mistakes by not touching the child - No Ebola cases in the community - Not trained on No-touch Policy - Other reason ________________________ |
| --- | --- | --- |
| 21 | Did you have any suspected or confirmed cases of Ebola infection in your community? | - Yes ❑ No |
| 22 | Did anyone die from Ebola in your community? | - Yes ❑ No |
| 23 | Do you know the symptoms of Ebola? | - Yes ❑ No |
| 24 | How did you learn about the symptoms of Ebola? | - Trained by Ministry of Health (in Month _______, 2014) - Trained by the Red Cross (in Month _______, 2014) - Trained by other NGO (in Month _______, 2014) - From media (TV, radio, posters, etc) - Other (Please state): ____________________________ |
| 25 | Have you diagnosed or suspected anyone with Ebola symptoms in your community? | - Yes ❑ No |
| 26 | If yes, what did you do? | - Nothing - Advised them verbally to go to the hospital or clinic - Referred them to the hospital or clinic (with a written paper) - Informed the Community Health Committee - Informed my supervisor or the Red Cross project staff - Others (please state) _____________________ |
| 27 | Did the local health facility or anyone from the MOH contact you on any issue during the Ebola response? | - Yes ❑ No |
| 28 | What kind of contacts did you have with health facility staff or others from the Ministry of Health? |  |
| 29 | Were you engaged in providing education to your community on how to protect themselves from Ebola? | - Yes ❑ No |
| 30 | If yes, what specific education activities did you provide? | - House-to-house visits - Community meeting - Other |
| 31 | Who supported you to provide these education activities?  *Multiple answer possible* | - Red Cross project staff - Health facility staff; MOH - Community Health Committee - Own initiative - Other |
| 32 | What actions did the Community Health Community take in response to the Ebola epidemic? |  |
| 33 | Did you have any contact with other Red Cross volunteers (e.g. CBHFAs) in your area on the Ebola epidemic? | - Yes ❑ No |
| 34 | If yes, what kind of contacts did you have? |  |
