# Supplementary material for: Community-based health care is an essential component of a resilient health system: evidence from Ebola outbreak in Liberia
Source: BMC Public Health. 2017 Jan 17;17:84. doi: 10.1186/s12889-016-4012-y (PMC5240441; doi:10.1186/s12889-016-4012-y)
Supplement: Additional file 4: — Categories and associated questions guiding the data analysis process. This is a summary of the broad categories and their associated questions used to analyze the data. (DOCX 16 kb) [file 12889_2016_4012_MOESM4_ESM.docx]

**Additional file 4**

**Categories and associated questions guiding the data analysis process**

Analysis of the data collected was carried out to document the trends over time in the community based service delivery by gCHVs supported by the Red Cross project, with the understanding that facility based services were suboptimal during the peak of the Ebola epidemic. The analysis sought to understand the reasons for the continuation and/or discontinuation of iCCM services at the community level. The analysis was guided by the following categories of analysis and their associated questions.

Community Case Management

- Was the coverage for community-based treatment of uncomplicated cases of child diarrhea and pneumonia sustained during the Ebola crisis?
- How were complicated cases of child illness and other cases for referral handled?
- What motivated CHVs to continue delivering health services in the midst of great personal risk?
- What factors contributed to continued service delivery by CHVs? (e.g. training, supplies, supervision by LRCS, etc.)

“No Touch” iCCM Guidelines

- What role did the introduction of the “No Touch iCCM” approach have in influencing the delivery of iCCM services by CHVs?
- Did training in the “No Touch iCCM” approach make a difference in CHVs confidence/delivery of services?

Additional Responsibilities of gCHVs

- Were there any additional services that the CHVs provided during the Ebola crisis that were not provided by them before the crisis? (e.g. services that were seen as essential and therefore provided by the CHVs due to the breakdown/overburdening of MoH facilities during the crisis)
- Did communities turn to the CHVs during the Ebola crisis, i.e. did they see them as health providers/skilled members of the community who could help?
- Did the CHVs engage with the communities to try to educate/prevent Ebola? Did the CHVs receive any additional training about Ebola prevention/response? If yes, from whom?
- What role did the Community Health Committees have during this time? Did they provide guidance and ensure appropriate oversight to CHVs activities?
- What impact did the lack of supervision by government health staff have on the CHVs?

Role of MNCH project

- What role did LRCS staff have during this time?
